# Electrophysiological Modeling and Molecular Dynamics Simulations of Cardiac Sodium Channels in Brugada Syndrome

**DOI:** 10.1155/bmri/4900599

**Published:** 2026-07-15

**Authors:** F. Shakibaei, S. H. Sabzpoushan

**Affiliations:** ^1^ Department of Biomedical Engineering, Iran University of Science and Technology (IUST), Tehran, Iran, iust.ac.ir

**Keywords:** action potential, brugada syndrome, cutoff radius, electric field, ionic strength, molecular dynamics, sodium ion channel

## Abstract

Brugada syndrome (BrS) is a rare but potentially fatal genetic cardiac disorder, primarily caused by mutations in sodium and potassium ion channels, leading to ventricular arrhythmias and sudden cardiac death. Despite advances in computational modeling, the precise effects of ionic environment and channel kinetics on BrS‐related action potentials remain incompletely understood. In this study, we developed a flexible electrophysiological model of cardiac sodium channels using a modifiable Richards activation function to simulate major BrS phenotypes and optimized the shape parameter (h) using particle swarm optimization (PSO). In parallel, molecular dynamics (MD) simulations were performed to analyze Na^+^ ion behavior in the selectivity filter under varying ionic conditions, providing molecular‐level insights into channel function. Results revealed that tuning the h parameter significantly improved key features of the action potential, including amplitude (APA), duration (APD), and time to peak (t_peak), aligning them more closely with physiological profiles. The protein structure was simulated in a solvated box using the AMBER03 force field under transmembrane potentials of 100–200 mV and at an ionic strength of 0.14 M. RMSD analysis confirmed greater structural stability of the protein in the presence of ionic strength; however, the additional ions created localized electric fields that initially disrupted ion flux. Increasing the applied voltage and cutoff radius to 1.4 nm reactivated ion transport, reproducing the “knock‐on” mechanism. Sensitivity analysis indicated that certain models exhibited stronger responses to changes in the activation function, highlighting their suitability for personalized modeling. The proposed model, without altering the fundamental channel structure, successfully simulates genetic dysfunctions by adjusting a single key parameter. It provides a practical framework for simulation‐based analysis of sodium channel dysfunction, arrhythmia risk assessment, and the exploration of personalized cardiac modeling strategies.

## 1. Introduction

Modeling the electrical behavior of cardiac cells is a powerful tool for analyzing the functional and pathological mechanisms of the heart. These models use mathematical descriptions to simulate membrane voltage (V) in response to electrical stimuli‐V changes driven by ionic currents passing through membrane channels, ultimately manifesting as action potentials. In electrophysiological studies, three complementary approaches are commonly employed: in vitro, in vivo, and in silico. The in vitro method investigates cellular behavior in a controlled environment outside the body, whereas in vivo experiments are conducted within living organisms and more closely mimic physiological conditions [1]. In silico modeling, through computational simulations, allows precise analysis at the molecular level [1].

Brugada syndrome (BrS) is a rare but potentially fatal genetic cardiac disorder characterized by ventricular arrhythmias and sudden cardiac death [2]. The primary genetic cause of this condition is mutations in the SCN5A gene, which encodes the Nav1.5 sodium channel—a critical component in the conduction of action potentials in cardiac muscle [3]. Understanding the structure and dynamics of these channels, particularly under varying biophysical conditions such as ionic strength, ion type, and electric field, is essential for elucidating disease mechanisms [4].

Among the cardiac arrhythmia syndromes associated with SCN5A mutations, BrS is selected as the primary disease model in this study due to its well‐established mechanistic link to loss‐of‐function alterations in the Nav1.5 sodium channel. This direct relationship makes BrS a suitable and clinically relevant framework for investigating sodium channel kinetics, ion permeation, and electrophysiological behavior using computational modeling and molecular dynamics (MD) simulations. Compared to other SCN5A‐related arrhythmic disorders with more heterogeneous mechanisms, BrS provides a more focused model for studying sodium channel dysfunction [2, 4, 5].

MD simulations, at the nanometric scale, offer a robust approach to investigating these factors. Ionic strength of the surrounding medium can influence channel stability, the organization of water and ions, and the gating mechanism. Alterations in this parameter may disrupt normal channel function. Combining MD simulation data with analysis of genetic mutations enables the development of targeted and personalized therapies for BrS patients.

Various studies have utilized functional assays, fluorescence techniques, and computational modeling to evaluate novel mutations [6–8]. Mutations in specific regions such as the voltage‐sensing domain (VSD), intracellular loops, or auxiliary subunits like *β*2 have been shown to reduce sodium currents (INas) and increase arrhythmogenic risk [9–11]. Some mutations also impair key protein–protein interactions, such as those between MOG1 and Nav1.5, reducing INa density [11]. Whole‐exome sequencing has identified pathogenic mutations in genes like SCN5A, NEBL, KCNQ1, and KCNH2—some associated with BrS and others with long QT syndrome [12]. Structural studies of SCN2B and SCN4B mutations further revealed differences in their impact on Nav1.5 function [13].

Investigations into the *β*2 subunit suggest that misfolding and defective glycosylation hinder proper trafficking and membrane localization of the sodium channel [14]. Clinical studies across diverse patient populations indicate that certain mutations (e.g., P/LP SCN5A) are more prevalent in young women of Caucasian descent and are associated with worse outcomes [15]. Intrinsically disordered regions (IDRs) have also been implicated in Nav1.5 functionality and mutation response [16]. Comprehensive analyses of various V‐gated ion channels have demonstrated that many arrhythmias are driven by mutations leading to prolonged action potentials [17].

Further simulation‐based studies have examined the kinetics of INa and its synergy with Phase 2 reentry (P2R)–related arrhythmogenesis [18]. Interdisciplinary research efforts aim to evaluate the translational power of bioinformatics tools, in vitro models, and animal studies for clinical application [19]. In silico modeling of human cardiac tissue fibers and loops has shown that spatial downregulation of sodium channels can induce notch‐and‐dome or loss‐of‐dome action potentials and promote reentry‐based arrhythmias, a hallmark of BrS pathophysiology [20].

Several specialized studies have advanced our understanding of BrS. In [21], the cellular basis of ST‐segment elevation was investigated using perfused RV wedge preparations, revealing that epicardial action potential dome loss leads to ST elevation and promotes P2R, ventricular tachycardia (VT), and fibrillation (VF); agents like isoproterenol and Ito blockers such as 4‐aminopyridine or quinidine normalized this effect. Recent advancements in artificial intelligence have also contributed significantly to BrS diagnosis.

AI has demonstrated the ability to detect subtle BrS ECG patterns and analyze stem cell‐derived cardiomyocyte data, often outperforming expert interpretation [22]. To address limitations in labeled datasets, a VICReg‐based self‐supervised deep learning model was developed in [23], achieving high diagnostic accuracy and uncovering previously underestimated prevalence rates. Moreover, a vision transformer (ViT) model in [24] predicted life‐threatening arrhythmic events from 12‐lead ECGs with high sensitivity and specificity, offering a novel tool for BrS risk stratification.

A deep learning ECG classifier presented in [25] demonstrated high diagnostic agreement (AUC = 0.96) compared to expert cardiologists and was validated across multiple centers in Asia. The electrophysiological mechanism underlying P2R in BrS was further explored in [1], emphasizing that dome loss alone is insufficient; specific profiles of delayed AP morphology and dome heterogeneity are required. Finally, [8] reported novel SCN5A gene variants in Polish patients with concealed BrS phenotypes, including SNPs affecting coding and noncoding regions, suggesting a potential link to hidden disease expression.

Despite these advances, the effects of ionic environment and channel kinetics on BrS‐related action potentials are not fully understood, and existing electrophysiological models often fail to capture key disease phenotypes. To address this, the present study develops a flexible electrophysiological model of cardiac sodium channels using a modifiable Richards activation function, optimizes the shape parameter (h) via particle swarm optimization (PSO), and integrates MD simulations to examine Na^+^ ion behavior in the channel selectivity filter under varying ionic conditions. By combining these approaches, we aim to provide mechanistic insights into how channel properties influence cellular electrophysiology in BrS and to establish a computational framework suitable for personalized modeling and arrhythmia risk assessment.

## 2. Method

### 2.1. Model Structure

This study is aimed at investigating the role of sodium channel gating velocity profiles in accurately simulating ion channel behavior and ionic current, as well as the impact of genetic mutations on diseases such as BrS. Initially, the behavior of ion channels and subsequently the ionic current are examined. The gating velocity profile of each gate represents the rate of gate opening at various membrane V values.

This profile determines the V range over which the ion channel activates faster or slower. To analyze the electrophysiological behavior of cardiac cells and assess the impact of these profiles on disease‐related characteristics, various computational models were employed. Mathematical models enable the study of cellular membrane properties and ion channel function under different conditions, and allow evaluation of how changes in biophysical parameters affect the electrical response of cells.

Both the established Hodgkin–Huxley (HH) electrophysiological model and a simplified version were utilized. These models employ nonlinear differential equations to describe membrane V dynamics and ionic currents; the modeling approach and the role of gating velocity profile functions within these frameworks are analyzed. Additionally, logistic and Richards mathematical functions were used to model the velocity profiles. The Richards function, by adding a flexibility h, provides greater adaptability to experimental data and abrupt changes in V‐dependent profiles, which is particularly important for analyzing the nonlinear behavior of sodium channels in BrS. This research focuses on examining sodium ion behavior in the channel′s selectivity filter region.

This region is entirely embedded within the protein structure and has no direct contact with the membrane; hence, removing the membrane does not significantly affect the local ion dynamics in this area. Therefore, the primary role of the membrane is limited to stabilizing the ion channel′s position within a specific spatial domain and preventing its free movement. Given that the speed and efficiency of MD simulation are directly related to the number of atoms in the system, the membrane was removed to optimize simulation performance. To compensate for the absence of the membrane and prevent free displacement of the protein, eight atoms within the structure (four in the upper region and four in the lower region) were fixed or “frozen” in their initial positions.

To initiate the simulation process, the atomic structure of the ion channel is required. This structure was obtained by retrieving the protein file with PDB code 1BL8 from the Protein Data Bank. This file contains comprehensive information about atom names and numbers, amino acid sequence (101 residues), and spatial coordinates of 1632 atoms in the protein structure. Next, the protein structure was placed inside a cubic simulation box with dimensions of 8.836 nm. To create an environment analogous to physiological conditions, the box was filled with 21,194 TIP3P water molecules. All simulation steps were performed using the Amber03 force field, which provides suitable parameters for sodium ions. Before starting the main simulation, the system was first energy‐minimized to reach a low‐energy stable state. Subsequently, the system was equilibrated in two separate phases: first, for 0.5 ns under constant temperature conditions (NVT ensemble) to stabilize the temperature; then, for 1 ns under constant temperature and pressure conditions (NPT ensemble) to maintain a pressure of 1 atmosphere.

### 2.2. Mathematical Formulation of the Model

#### 2.2.1. Ion Channel Gating Velocity Profiles

##### 2.2.1.1. Logistic Function

The logistic function is a sigmoidal (S‐shaped) function widely used to model processes exhibiting saturating or nonlinear behavior. The general form of the logistic function is as follows:

(1) mv=11+expv12−v/k.

In the equations, the function *m* represents the gating variable indicating the degree of ion channel opening, which is a function of the membrane voltage *v*. Here, *v* is the independent variable, and the parameter *v*_12_ denotes the V at which the ion channel gate is half‐open. The parameter *k* represents the maximum (or minimum) slope of the function. In ion channel modeling, this function is used to describe the probability of gate opening or closing as a function of membrane V. In this study, to achieve greater flexibility in modeling nonlinear variations and the effects of genetic mutations, the Richards function—a generalization of the logistic function—was employed.

##### 2.2.1.2. Richard′s Function

The Richards function is a generalized form of the logistic function that includes an additional parameter to control the shape of the curve. It is defined as follows, where:

(2) mv=11+h.exphvinr−v/kr1/h,

where *h* is the shape parameter of the function (when equal to 1, the function reduces to the logistic function), *v*_*i* *n* *r*_ represents the V corresponding to the point of maximum gating opening rate, and *k*_*r*_ is the slope parameter in the Richards function. Compared to the logistic function, the Richards function offers greater flexibility and is better suited for more accurate modeling of gating velocity profiles.

#### 2.2.2. Employed Models

##### 2.2.2.1. HH Model

**Figure 1:**
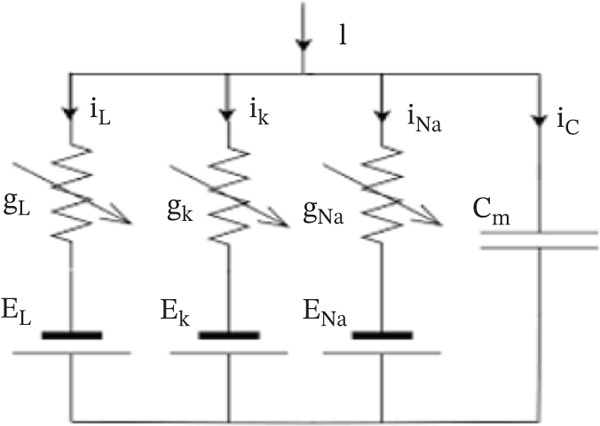
Equivalent circuit of the Hodgkin‐Huxley neuron model [26].

The HH model (1952) is recognized as the gold standard for computational models of the nervous system. In Figure 1 this model, a neuron is represented as a theoretical RC circuit: the cell membrane is modeled as a capacitor, whereas ion channels are treated as variable conductors. The conductance of these channels depends on factors such as membrane V, ionic currents, and ion concentrations. To describe these interactions, Hodgkin and Huxley derived a set of differential equations that account for three primary conductance sodium, potassium, and leak currents. This conductance is regulated by gating variables that depend on the cellular conditions [27–29].

The first model of mammalian cardiac cells was developed in 1962 by Denis Noble. This model closely resembled the HH model in terms of the involved currents and conductance. However, the Denis Noble model incorporates a slow potassium current. Due to the slow activation of this secondary current, the leakage current can spontaneously and regularly depolarize the cells without the need for external stimulation. In addition, baseline conductance and equilibrium potential were modified. Finally, there were slight changes in the gating kinetics that govern neuronal behavior [26, 30].

(3) Itotal=gNam3hV−ENa+gkn4V−Ek+gLeakV−ELeak.

For each gating variable:

(4) dXdt=αxX1−X−βx,

where *v* is membrane potential (mV), *C*_*m*_ is membrane capacitance (*μ*F/cm^2^), *I*_*N* *a*_ is fast INa, *I*_*K*_ is fast potassium current, *I*_*L*_ is leak current, *m*, *n*, *h* channels being open or closed, and *α*, *β* is V‐dependent rate functions for the opening and closing of each gate, originally determined empirically in the HH paper.

##### 2.2.2.2. *I*_*N* *a* *p*_ + *I*_*K*_ Model

The model is a simplified model that includes fast INa and potassium current. It is well‐suited for initial analyses of action potentials and is used for more detailed investigations of the effects of genetic mutations and cardiac disorders such as BrS. This minimal two‐variable model effectively reproduces many key features of the action potential [31]. Therefore, for an initial evaluation of the hypothesis proposed in this study, it will be used as the action potential model. This model is considered one of the most fundamental and useful models in neuroscience. It includes a fast INa and a relatively slower potassium current. The main equations of this model are as follows:

(5) v.=−gLv−EL−gNamvv−ENa−gknv−Ek

(6) n.=1τnnv−n

(7) nv=11+expv12n−v/kn

(8) mv=11+exp v12m−v/km 

The parameter *m*(*v*) depends on whether the logistic or Richard′s function is used in the simulation. Conductances *g*_*N* *a*_, *g*_*K*_, *g*_*L*_ and equilibrium potentials *E*_*N* *a*_, *E*_*K*_, *E*_*L*_ are also denoted using their respective standard symbols. The model includes two activation functions: one for sodium ions and one for potassium ions, either of which—or both—may undergo alterations due to genetic mutations. It is evident that, in this specific case, the sodium gate is instantaneous and lacks dynamic behavior, primarily influencing the upstroke phase of the action potential, whereas the potassium gate is more dynamic and affects the temporal characteristics of the membrane potential.

### 2.3. Model Parameter Tuning

The proposed model in this study includes only one tunable parameter: the shape parameter ℎ in the Richards activation function. Other parameters in the function were randomly selected within biologically and logically valid ranges. The coding required for simulation and optimization was implemented using MATLAB. The parameter ℎ plays a critical role in defining the shape of the activation rate profile of ion channels. It controls the asymmetry of the activation curve and directly affects the excitability of the cell membrane one of the key features in the electrophysiological performance of cardiac cells. To evaluate the impact of this parameter, simulations were conducted across various values of ℎ within the employed models. The results of these simulations are presented and analyzed in subsequent sections.

One of the advanced and widely used tools in analyzing the behavior of biomolecules is MD simulation. This method is based on solving Newton′s equations of motion for the atoms and molecules within a system, allowing researchers to predict and analyze its dynamic properties over timescales ranging from nanoseconds to microseconds. A primary challenge in biomolecular simulations lies in calculating long‐range electrostatic interactions between particles, as these calculations are highly demanding and complex due to their dependence on spatially distant interactions.

To optimize this process, the Ewald Summation method—particularly the Particle Mesh Ewald (PME) variant—is employed. In this technique, each charged particle is paired with an opposite Gaussian‐distributed charge, allowing the total electrostatic potential to be decomposed into two computationally manageable components: real space and reciprocal (Fourier) space. By appropriately tuning parameters such as the Gaussian distribution width, the convergence rate of the two series can be improved, thereby significantly reducing the computational time.

A critical assumption in applying this method is that the net charge of the system must be neutral. To satisfy this condition, two main strategies are commonly used: (1) the addition of counter‐ions to ensure overall charge neutrality, and (2) adjusting the ionic strength of the environment to mimic physiological conditions. Ionic strength is a key parameter in simulating biological environments and is calculated using the following relation:

(9) I=∑iCiZi,

where *C*_*i*_ represents the ionic concentration (in molarity), *Z*_*i*_ is the charge of ion *i*, and *i* *i* *i* indexes the different types of ions present in the system.

To assess the stability of the protein′s three‐dimensional structure during the simulation, the root mean square deviation (RMSD) index was used. RMSD quantifies the deviation of the current structure from the initial conformation and is calculated using the following equation:

(10) RMSDt1,t2=1M∑i=1Nrit−ri0212/,

where *m*_*i*_ is the mass of atom *r*_*i*_, *i* is the instantaneous position of this atom at time *t*, and *M* is the total mass of the system.

Moreover, RMSD serves as a key metric for evaluating the structural stability of the biomolecular system and investigating the impact of environmental parameters on the studied protein conformation.

### 2.4. PSO Algorithm

In this study, PSO was employed to optimize the parameter *h* and achieve the best fit between the simulated models and the natural action potential profiles. PSO is a population‐based optimization algorithm inspired by the collective behavior of natural organisms such as birds or fish. In this method, each particle moves through the search space, continuously updating its position based on its own best experience and that of its neighbors to approach the optimal solution.

In the present work, the PSO algorithm was used to optimize the parameters of various cardiac action potential models and was capable of effectively tuning model parameters. The results demonstrate that the application of PSO significantly improves the accuracy of simulations and enhances their agreement with experimental data. Moreover, this algorithm successfully identifies optimal values for the Richards function parameters, which can potentially mitigate the effects of BrS and restore pathological states toward normal physiological conditions.

The objective of the optimization algorithm was to minimize model errors and maximize alignment with the normal physiological behavior of cardiac cells. The optimal value of the shape parameter *h* served as a key indicator; when applied, it led to the greatest reduction in the discrepancy between the electrical characteristics of the diseased and normal states.

## 3. Results

### 3.1. Model Simulation

This section presents two categories of results. The first category includes the simulation outcomes of cardiac action potentials using classical electrophysiological models, specifically the HH and *I*_*N* *a* *p*_ + *I*_*K*_ models. These results are provided for three physiological conditions: (i) normal state, (ii) BrS Type I, characterized by a potassium channel dysfunction, and (iii) BrS Type II, involving a sodium channel defect.

The second category focuses on molecular modeling and structural analysis of the NaV1.5 sodium channel under different ionic strength environments. This includes evaluations of electrostatic fields, cutoff radius parameters, and the knock‐on conduction mechanism. Additionally, emphasis is placed on the role of the physiological environment and its influence on sodium ion permeation through the channel.

In the first part of the simulation, the impact of the Richards activation function and variations in its shape parameter (h) at values of 0.3, 1, and 1.3 were investigated. The results were analyzed to understand the formation of action potentials under both normal and pathological conditions, assess the effect of parameter *h* on restoring physiological features, and evaluate the efficiency of the PSO algorithm in accurately tuning this parameter. Particular focus was placed on changes in action potential amplitude (APA), action potential duration (APD), and time to peak (t_peak) in response to optimization of the Richards function.

The simulation outcomes were interpreted based on physiological characteristics: in BrS Type I, a reduction or loss of the action potential dome in the epicardial layer, due to increased potassium current or significant INa reduction, leads to accelerated repolarization and a notable V gradient between the epicardium and endocardium. Conversely, in Type II, the action potential dome is maintained but with reduced amplitude and slope, reflecting only mild INa impairment [32].

#### 3.1.1. Results Obtained From the *I*_*N* *a* *p*_ + *I*_*K*_ Model

**Figure 2:**
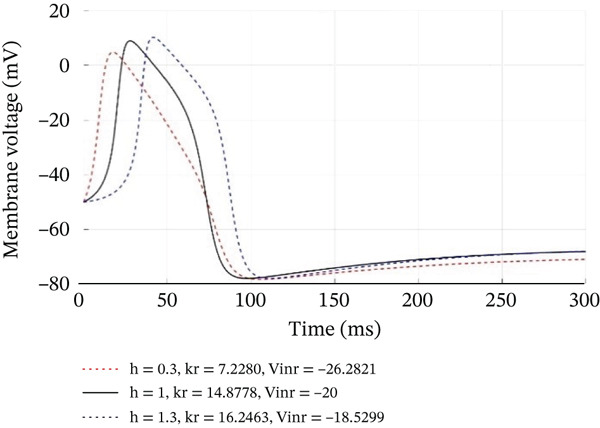
Graph of membrane potential changes over time for different values of *h* in the *I*_*N* *a* *p*_ + *I*_*K*_ model with the Richards activation function applied to the *N* *a* coefficient.

In Figure 2, with increasing values of the shape parameter *h* in the Richards activation function for *g*_*N* *a*_, the time to reach the peak potential decreases, and the amplitude of the potential increases. This indicates a faster depolarization and a stronger membrane response to stimulation.

**Figure 3:**
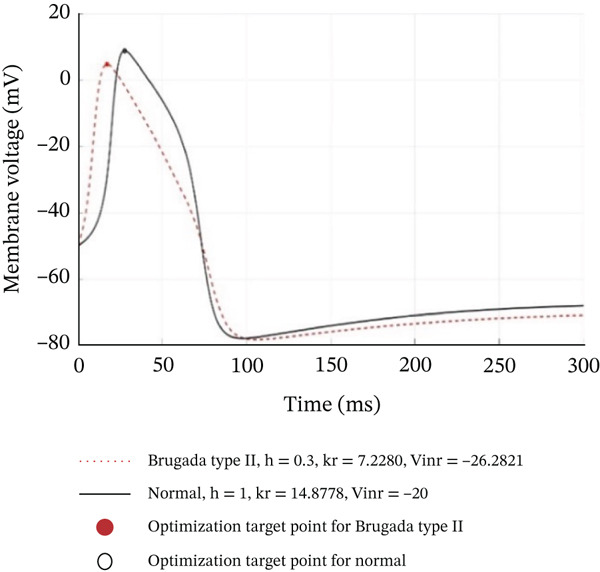
Graph of the *I*_*N* *a* *p*_ + *I*_*K*_ model with Richards activation function applied to the *N* *a* coefficient for optimizing the Brugada Type II condition to normal.

In Figure 3, by optimizing the parameter *h* for *g*_*N* *a*_ under Brugada Type II conditions, the amplitude and duration of the action potential return to values close to the normal state, indicating the high effectiveness of this function in restoring membrane dynamics.

**Figure 4:**
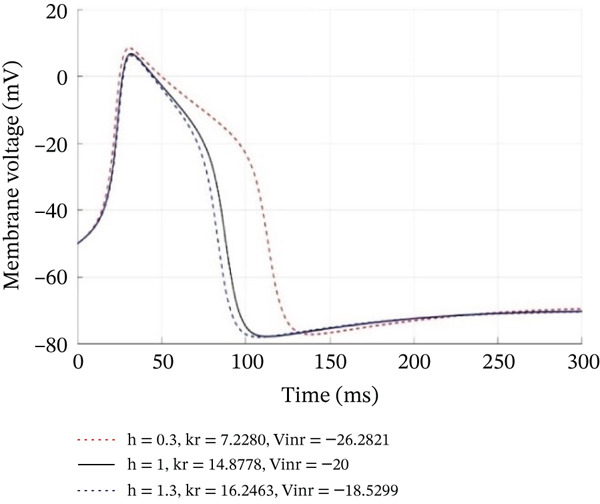
Graph of membrane potential changes over time for different values of *h* in the *I*_*N* *a* *p*_ + *I*_*K*_ model with the Richards activation function applied to the potassium coefficient.

In Figure 4, with the increase of *h* in the *g*_*N* *a*_ activation function, the repolarization process is modulated and the duration of the phase is extended.

**Figure 5:**
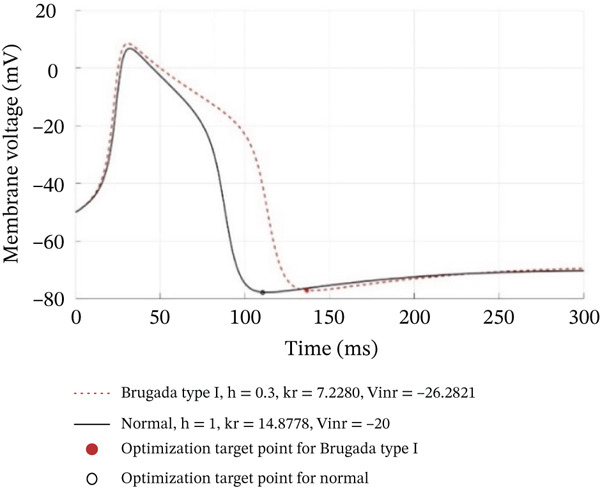
Graph of the *I*_*N* *a* *p*_ + *I*_*K*_ model with the Richards activation function applied to the K conductance for converting Brugada Type I condition to normal.

In Figure 5, optimization of the parameter *h* under Brugada Type I conditions resulted in the restoration of the action potential waveform to its normal state. The results showed that increasing the value of *h* led to an increase in the APA and a decrease in its duration (APD). This effect was especially notable in BrS Type II (caused by sodium channel dysfunction), where the waveform was restored to normal. The PSO optimization algorithm successfully adjusted the value of *h* to minimize the difference between the pathological and physiological states.

#### 3.1.2. Results Obtained From the HH Model

**Figure 6:**
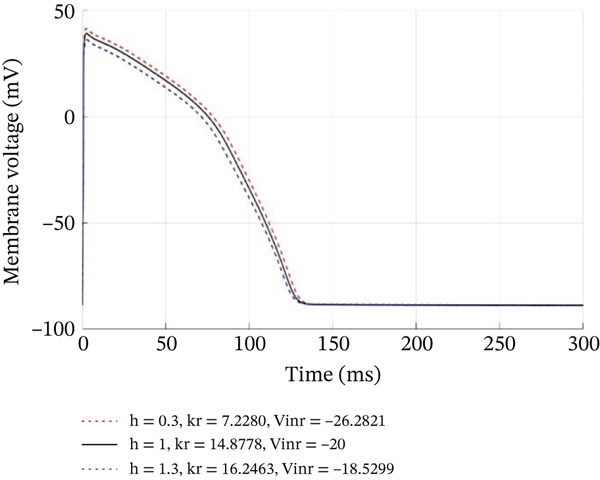
Graph of membrane potential changes over time for different values of *h* in the Hodgkin–Huxley model with the Richards activation function applied to the Na coefficient.

In Figure 6, increasing *h* in *g*_*N* *a*_ causes an increase in the speed and amplitude of depolarization.

**Figure 7:**
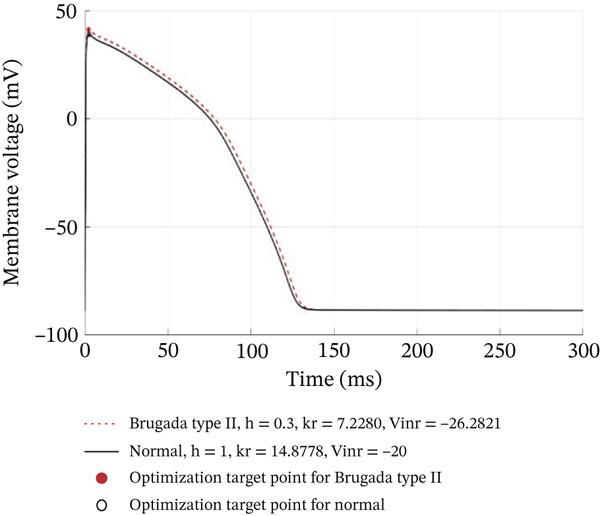
Graph of the Hodgkin–Huxley model with the Richards activation function applied to the Na coefficient for optimizing the Brugada Type II condition to normal.

In Figure 7, optimizing the parameter *h* in Type II BrS led to an improved action potential shape, bringing it closer to the normal state.

**Figure 8:**
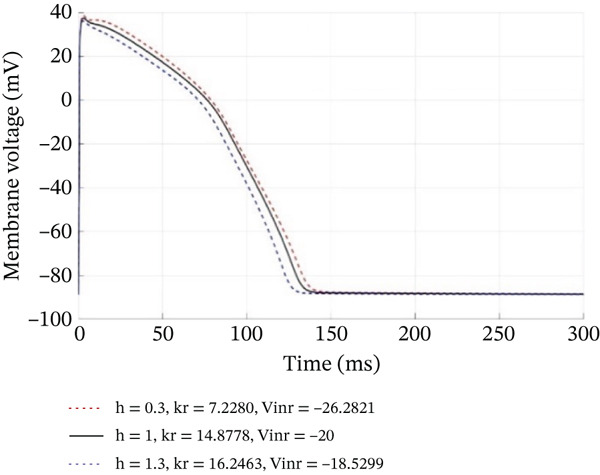
Graph of the Hodgkin–Huxley model with the Richards activation function applied to the K coefficient.

**Figure 9:**
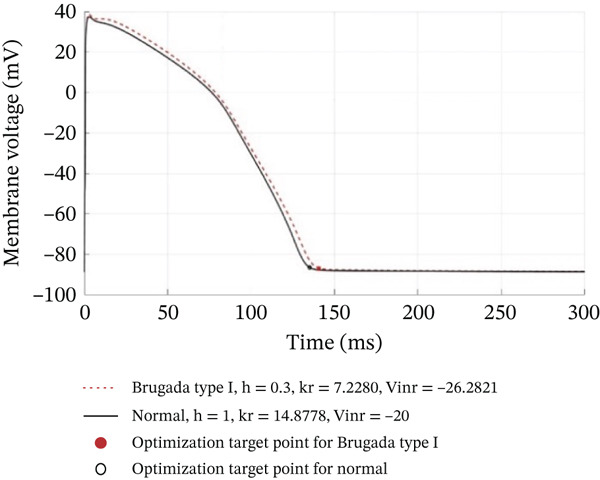
Graph of the Hodgkin–Huxley model with the Richards activation function applied to the K coefficient for optimizing the Brugada condition to normal.

In Figures 8 and 9, variations of *h* in *g*_*k*_, similar to other models, led to improvements in the repolarization phase and correction of the membrane response under pathological conditions. Simulations of the HH model showed that changes in the Richards activation function applied to the INa also increased the amplitude of depolarization and decreased t_peak. The application of the PSO algorithm to optimize parameter *h*, especially in BrS Type II, resulted in significant improvements in peak V and the overall shape of the action potential. Sensitivity analysis in this model indicated a moderate responsiveness to parameter *h*, with APA changing by approximately 2.32 mV and APD by about 5.48 ms. These values are lower than those in other models, reflecting the relative stability of the HH model against variations in the activation function.

### 3.2. Ionic Strength Analysis

This paper attempts to study key parameters in the MD simulation of the sodium ion channel, which is a key unit for the movement of ions through cell membranes. Key parameters include ionic strength, electric potential, and the cutoff radius for van der Waals and Coulombic interactions. Whereas the sodium ion channel is itself a membrane protein, the lipid membrane was left out of consideration in the simulation to reduce computation load as the earlier work has shown no significant effect of the membrane on sodium ion passage. The focus has been put upon the selectivity filter region of the channel that lies within the protein and remains unaffected by the membrane. To avoid free movement of the protein once the membrane was removed, eight atoms of the protein structure were anchored in position.

**Figure 10:**
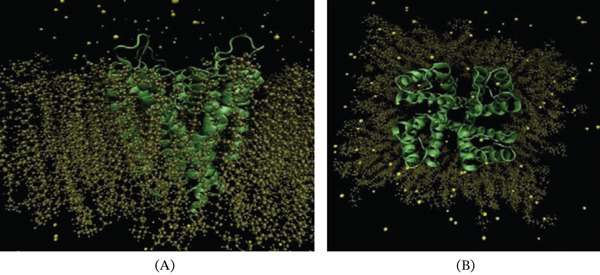
Top view (A) and side view (B) of the sodium ion channel in the bilayer membrane.

The simulation process is started by retrieving the atomic structure of the sodium ion channel (Code 1BL8) from the protein database, and it provides minute information about atoms, amino acid sequence, and spatial coordinates. The protein is then placed inside a cubic simulation box (8.836 nm) containing 21,194 TIP3P water molecules. Amber 03 force field is used for simulation, which is suitable for sodium ions. Before the main simulation, energy minimization is performed, followed by two equilibration phases: temperature stabilization at first (NVT ensemble) for 0.5 ns, followed by pressure stabilization (1 atm) in isothermal‐isobaric conditions for 1 ns. Radii of Coulomb and van der Waals interactions are set to 1.2 nm, and an electric potential of 100 mV is assigned between the channel so as to simulate physiological conditions. As shown in Figure 10:

One hundred millivolts of electric potential difference are applied in the simulations between the 3.4 nm long sodium ion channel. The potential difference is modeled as a constant electric field along the *z*‐axis (the channel′s central axis), with a magnitude of 29.41 mV/nm. To explore how ionic strength affects the dynamic activity of sodium ions in the channel, several simulations under different conditions were conducted. In the first simulation, considered as the baseline or reference state (designated as I), only negatively charged chloride ions (*C* *l* ^−^) were added solely to neutralize the overall charge of the system. Under this condition, the ionic strength approaches zero, and only the effects of the equilibrium ions are investigated.

**Figure 11:**
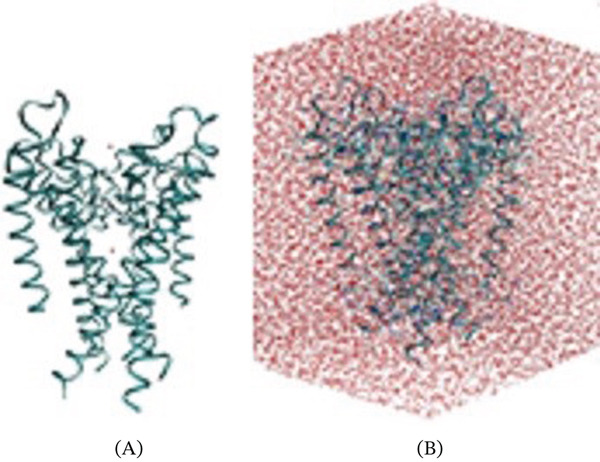
(A) Ion channel structure. (B) Ion channel in the simulation box.

**Figure 12:**
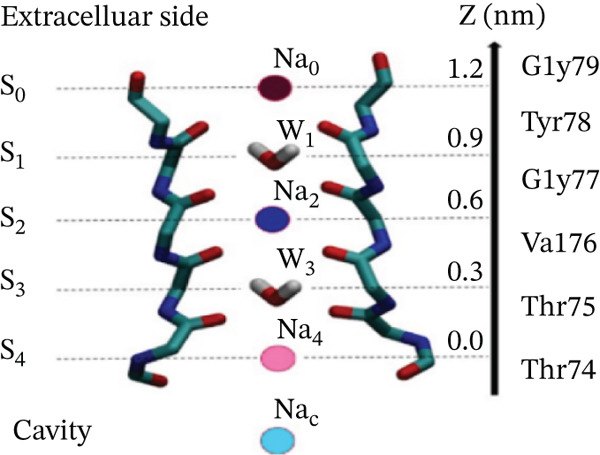
Location of sodium ions inside the selector filter before the simulation begins.

In these simulations, the goal is to determine the role of environmental electrolytes in the passage of ions through the sodium channel selectivity filter by comparing the behavior of ions under different ionic strengths and to gain a better understanding of the ion transport mechanism under physiological and unusual conditions; As shown in Figures 11 and 12:

(11) I=12nCl−+mNa+181000×number of water/nCl−+mNa+=0

Here, to simulate the physiologic conditions of living organisms, the solution’s ionic strength is created approximately 0.14 molar (I = 0.14 M). For this, a specific quantity of negative chloride ions (*C* *l* ^−^) and positive sodium ions (*N* *a* ^+^) are added to the system with an equation where *n* represents the quantity of chloride ions and *m* the quantity of sodium ions. This combination of ions not only removes the net charge of the system but also sets up the desired ionic strength. This new state, where the ionic strength is taken into account, is then denoted by the symbol  ^′^ *I*.

At this stage, the system is set for a 10‐ns constant pressure and temperature (NPT ensemble) MD simulation to investigate the sodium ion dynamics within the ion channel. The setup places the *N* *a*_*c*_ ion at the center cavity, *N* *a*_4_ at the *S*_4_ site, *N* *a*_2_ at the *S*_2_ site, and *N* *a*_0_ at the *S*_0_ site, and water molecules *W*_1_ and *W*_3_ at *S*_1_ and *S*_3_ to keep the structure stabilized.

In the traces shown in Figure 4a (state I, zero ionic strength), the *N* *a*_0_ ion exits and departs from the channel at the onset. The *N* *a*_*c*_  ion shifts to the *S*_4_ position, and *N* *a*_4_ shifts to *S*_3_. *N* *a*_2_ shifts from *S*_2_ to *S*_1_ and exits the channel around 1 ns. This set of shifts shows ion displacement within the channel. The ion passage rate is approximately 10^7^ to 10^8^ ions per second, as seen in experimental results.

**Figure 13:**
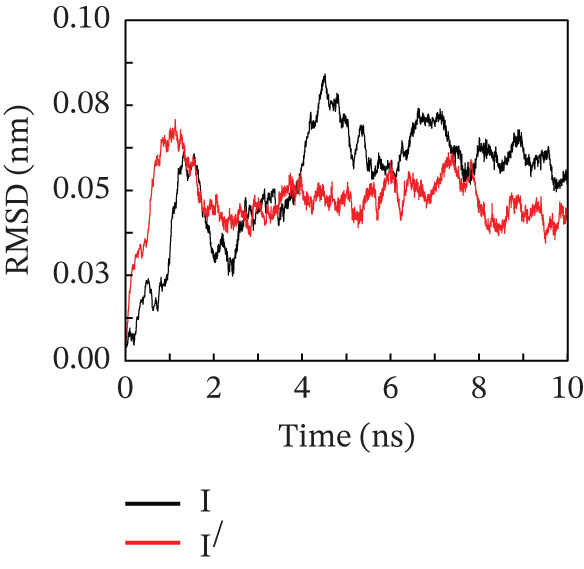
Behavior of sodium ions over time at a potential difference of 100 mV under conditions I and I ^′^.

**Figure 14:**
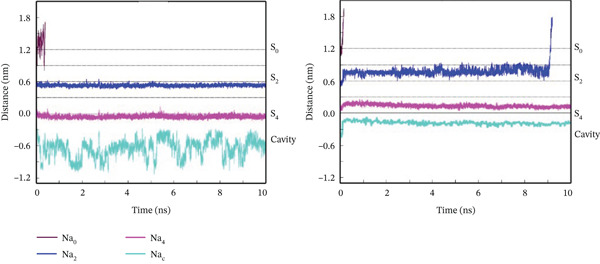
RMSD graph related to the normal simulation (black) and the simulation in the presence of ionic strength (red).

As opposed to that, Figure 13b depicts state I ^′^ results where only the simulation is performed at 0.14 molar ionic strength. In this case, only the *N* *a*_0_ ion, which originates from *S*_0_, moves even farther away from its original position, but other ions like *N* *a*_*c*_, *N* *a*_4_, and *N* *a*_2_  remain in their original positions with just slight oscillations without reaching up to the next positions. As a result, the ion flow process is halted, and no effective ion passage via the channel is observed under these conditions. To further investigate the structural stability and atomic position changes of the protein in the two simulation states, the RMSD plot is given in Figure 5. The plots give dynamic difference information of the channel structure with or without ionic strength and useful information on the stability and activity of the channel. As shown in Figures 13 and 14.

The observation is made by the analysis that the sodium ion channel structure in condition I ^′^ (ionic strength, in red) is more stable than in condition I (no ionic strength, in black). This indicates that considering ionic strength increases the validity and accuracy of MD simulations. However, it also induces disarrangements in the ion flux.

The existence of other ions, for example, *N* *a* ^+^ and *C* *l* ^−^, that were used to neutralize the system creates a local electric field. The electric field opposes the natural potential difference across the membrane, causing an interruption in the movement of the ions. It has been proven by previous research that there exist certain regions on the protein surface where ions can become trapped, inhibiting their movement for extremely long times (hundreds of picoseconds). This generates a localized electric field that slows the ions′ random motion by a factor of about one‐third.

**Figure 15:**
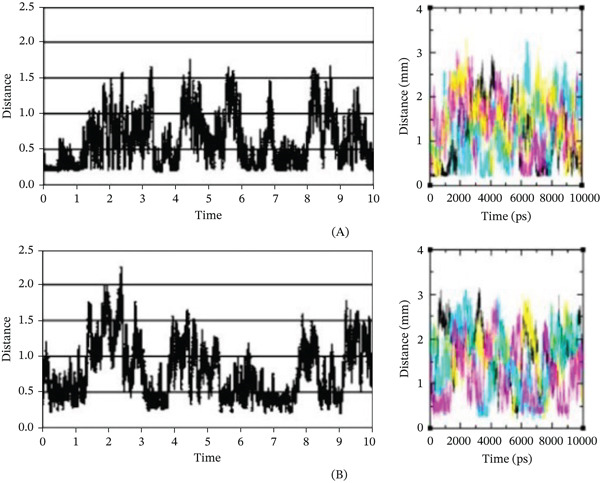
Dynamics of (A) a *C* *l* ^−^ ion and all *C* *l* ^−^ ions and (B) a Na^+^ ion and all Na^+^ ions [15].

**Figure 16:**
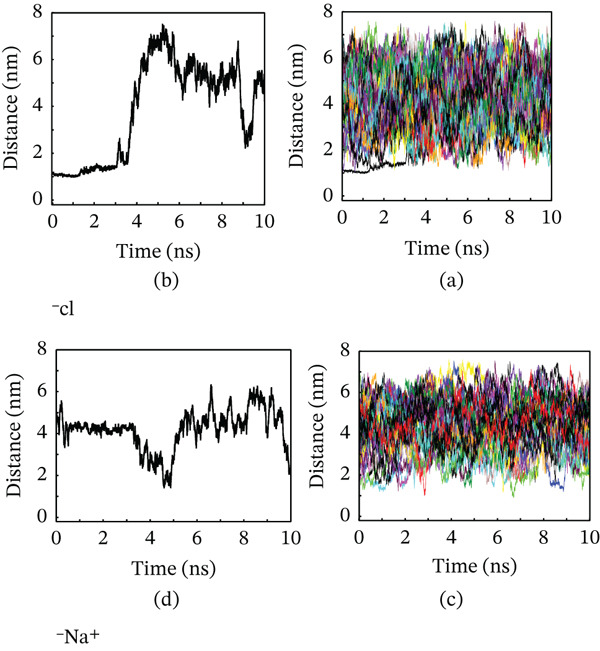
Dynamics of (a) all *C* *l* ^−^ ions, (b) one *C* *l* ^−^ ion, (c) all *N* *a* ^+^ ions, and (d) one *N* *a* ^+^ ion in Conditions: I = 0.14 M and potential difference = 100 mV.

**Figure 17:**
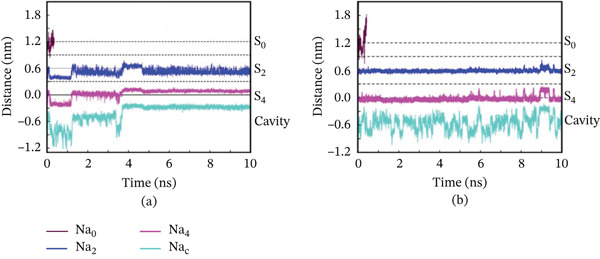
Behavior of sodium ions over time under Conditions *I* = 0.14 M and potential difference: (a) 150  and (b) 200 mV.

Figure 15A,B illustrates the limitation of ion mobility (*C* *l* ^−^ and *N* *a* ^+^) within some time ranges, that is, between 0 and 1 ns and 7 and 8 ns for *C* *l* ^−^ ions, and between 2.3–3.8 ns and 4.5–7.6 ns for *N* *a* ^+^ ions. The right panels of the figures show the general dynamics of *C* *l* ^−^ and Na^+^ ions. As shown in Figure 15:

In the simulation conducted at an ionic strength of 0.14 M and a potential difference of 100 mV, the movement of *N* *a* ^+^ and *C* *l* ^−^ ions showed similar results to those in reference [15]. A *C* *l* ^−^ ion mobility was restricted between 0 and 3 ns, and a Na^+^ ion showed restricted mobility between 0.1–3 ns and 8–8.8 ns. This is because the local electric field, created by the neutralizing ions, opposed the natural electric field across the membrane, disrupting ion flow. Attempts to negate this by strengthening the electric field (to 14.71 mV/nm and 29.41 mV/nm) still resulted in no ion flow, as seen in Figures 16 and 17 below.

**Figure 18:**
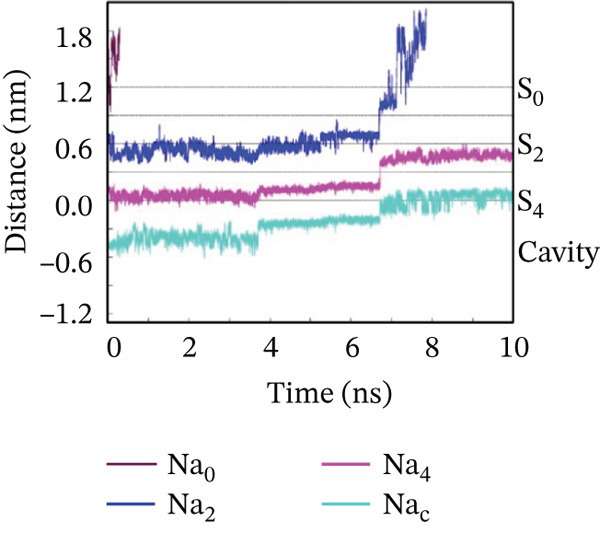
Behavior of sodium ions over time at a potential difference of 200 mV and conditions of *I* = 0.14 M,

**Figure 19:**
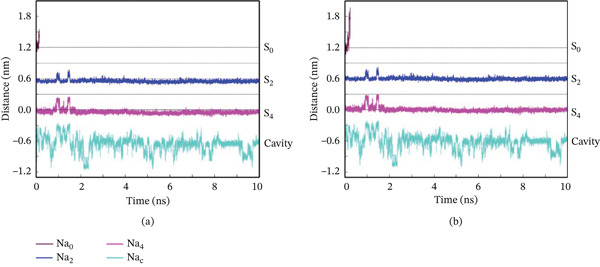
Behavior of sodium ions over time at a potential difference of –100 mV under Conditions (a) I and (b) I ^′^.

**Figure 20:**
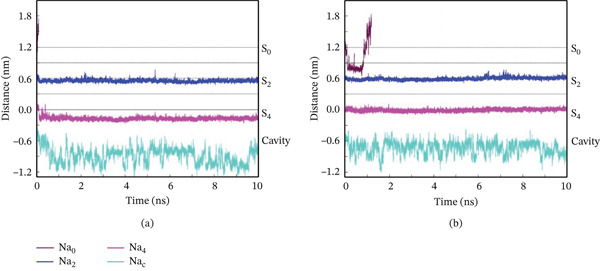
(a,b) Sodium ion trajectories at 0 mV under Condition I (no ionic strength), illustrating the absence of ion conduction due to insufficient driving force.

In the next step, simultaneously with increasing the field to 58.82 (equivalent to a potential difference of 200 mV), we increased the cutoff radius from 1.2 to 1.4 nm. As shown in Figure 9, the ion flow is well observed according to the “knock‐on” mechanism. So that initially, the ion located in the cavity approaches the filter′s mouth and pushes the two ions located in the filter, eventually causing the ion at the top of the filter to exit the channel. Considering that taking into account the ionic strength, changing the potential difference, and changing the cutoff radius affects the results obtained from the simulation, it was decided to examine the behavior of sodium ions within the selective filter at radii of 1.2, 1.4, and 1.6 nm and different potential differences of 0 0, −100, 100, and 200 mV once with ionic strength and once without applying ionic strength. As shown in Figures 18, 19, and 20

′

#### 3.2.1. Review of Different Cutoff Radii

##### 3.2.1.1. Beam Cut nm 1/2

At a potential difference of 100 mV under Condition I (Figure 19a and under condition I ^′^ (Figure 19b), as well as at a potential difference of 0 mV under condition I (Figure 20a) and under condition I ^′^ (Figure 20b), no flow occurs.

**Figure 21:**
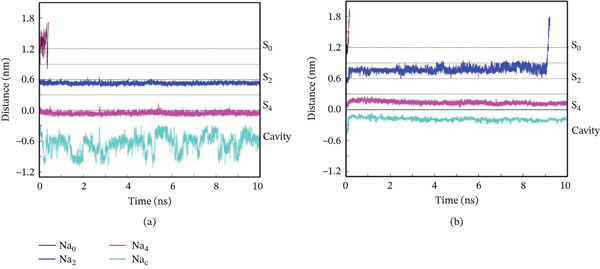
(a,b) Sodium ion movement at 100 mV under Conditions I and I ^′^, comparing ion transport with and without ionic strength.

**Figure 22:**
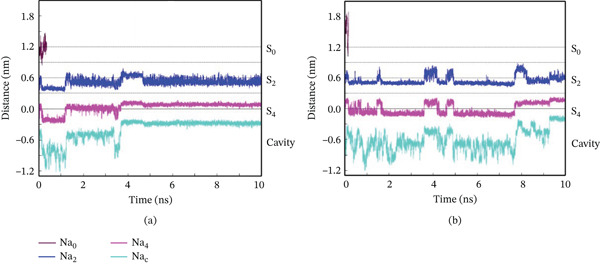
Behavior of sodium ions over time at a potential difference of 200 mV under Conditions (a) I and (b) I ^′^.

As seen in Figure 21a, under a potential difference of 100 mV and conditions I, meaning without considering ionic strength, the *N* *a*_0_ ion located at position *S*_0_ is released immediately after the simulation begins. The *N* *a* ^+^ ion moves from the cavity to position *S*_4_, and the *N* *a* ^+^ ion moves from position *S*_4_ to position *S*_3_. The Na^+^ ion first moves from position *S*_2_ to position *S*_1_ and exits the channel after 9 ns. According to Figure 21b, at a potential difference of 100 mV and under condition I ^′^, sodium ions are unable to leave their initial positions, resulting in disrupted ion flow. As shown in Figure 21:

In a 200‐mV potential difference and condition I (Figure 22a), within the time frame of 3 to 5 ns, the ions located at positions *S*_4_ and *S*_2_ move from their initial positions to the next positions but then return to their initial positions. Under the same potential difference and conditions, I ^′^ (Figure 22‐b), the ions have jumps in their positions but are unable to move to the next positions, resulting in no ion flow. As shown in Figure 22:

After examining the behavior of sodium ions at a cutoff radius of 1.2 nm and different potential differences under conditions I and I ^′^, we will investigate their behavior at a cutoff radius of 1.4 nm and subsequently at a cutoff radius of 1.6 nm.

##### 3.2.1.2. Beam Cut nm 1/4

**Figure 23:**
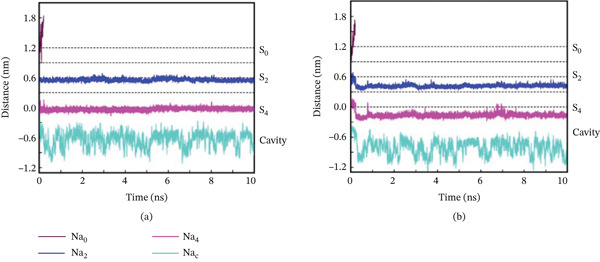
Behavior of sodium ions over time at a potential difference of −100 mV under Conditions (a) I and (b) I ^′^.

At a potential difference of −100 mV and under condition I, as shown in Figure 23a, the ions located in the cavity, positions *S*_4_ and *S*_2_, move in the direction of the applied electric field from the time interval of 0–0.4 ns and reach their downstream positions, where they continue to oscillate until the end of the simulation. Under condition I ^′^ as shown in Figure 23b, only the ion located at the outermost part of the selective filter, namely the *N* *a*_0_ ion at position *S*_0_, exits the channel at the start of the simulation, whereas the other ions remain in their initial positions. As shown in Figure 23.

**Figure 24:**
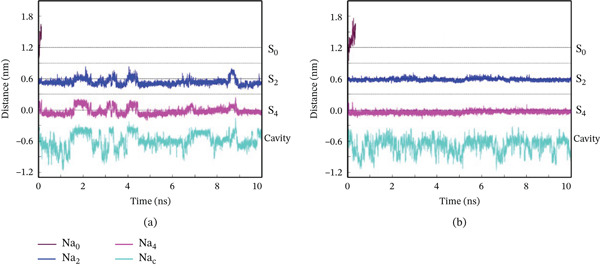
(a, b) Sodium ion behavior at 0 mV under Condition I (with ionic strength), highlighting the influence of localized electric fields on ion mobility.

**Figure 25:**
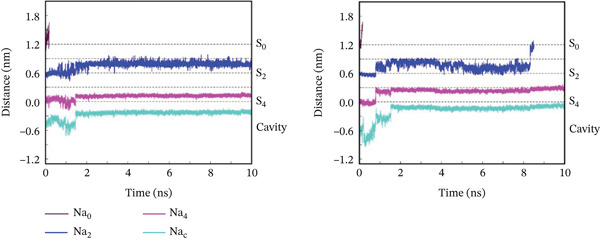
Sodium ion trajectories at 100 mV and cutoff radius of 1.4 nm, demonstrating restored ion conduction under optimized conditions.

**Figure 26:**
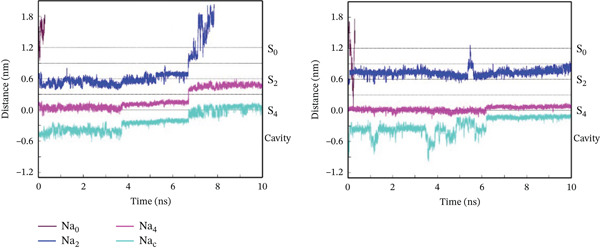
Sodium ion trajectories at 200 mV under Conditions I and I ^′^, illustrating the reactivation of ion conduction via the knock‐on mechanism at higher electric potential.

At a potential difference of 0 mV and under condition I, as shown in Figure 24a, only the *N* *a*_0_ ion located at position *S*_0_ exits the channel, and the other three ions are unable to leave their initial positions; thus, no flow occurs. Under condition I ^′^, as shown in Figure 24b, in addition to the exit of *N* *a*_0_ ions, *N* *a*_4_, *N* *a*_2_, and *N* *a*_3_ ions are also able to move during certain time intervals. Considering the zero value of the main electric field, one can observe the trace of a localized electric field caused by the presence of neutralizing ions. As shown in Figure 24:

In the simulations, with a potential difference of 100 mV and condition I (where we did not consider ionic strength), there was ion flow. The *N* *a*_0_ ion was released, and the subsequent ions (*N* *a*_*c*_, *N* *a*_4_, and *N* *a*_2_) moved through the channel, with *N* *a*_2_ exiting after 8.5 ns, thus leading to ion flow. However, when we used condition I ^′^ (where we considered the ionic strength), its sodium ions did not move but remained in place, thus there was no ion flux. No movement of ions was observed at 200 mV and condition I. At 200 mV and condition I ^′^, ions in the cavity, *S*_4_, and *S*_2_ sites moved at 3.8 ns and remained at the new positions for 3 ns. These ions persisted in moving by 8.6 ns, and the *N* *a*_2_  ion exited the channel, successfully accomplishing ion flow via the “knock‐on” mechanism. As shown in Figures 25 and 26.

##### 3.2.1.3. Beam Cut nm 1/6

**Figure 27:**
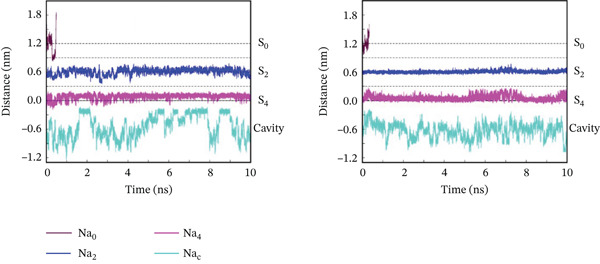
Behavior of sodium ions over time at a potential difference of −100 mV under Conditions I and I ^′^

**Figure 28:**
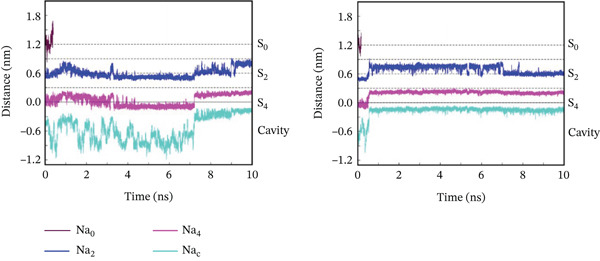
Sodium ion dynamics at 0 mV with a cutoff radius of 1.6 nm under both I and I ^′^ conditions, showing negligible ion transport.

**Figure 29:**
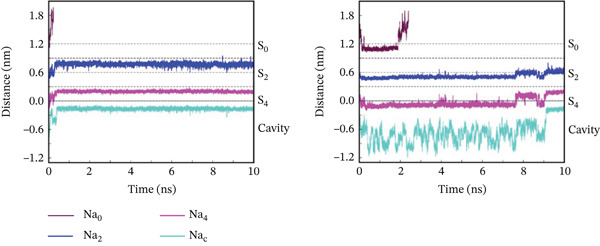
Sodium ion behavior at 100 mV with a cutoff radius of 1.6 nm, indicating reduced sensitivity of ion flow to increased cutoff distance.

**Figure 30:**
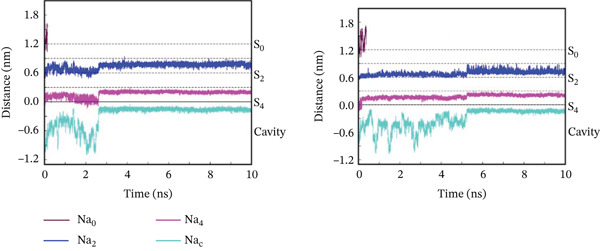
Behavior of sodium ions over time at a potential difference of 200 mV under Conditions I and I ^′^.

As shown in Figures 27, 28, 29 and 30 considering that the simulations conducted at a cutoff radius of 1.6 nm and different potential differences under conditions I and I ^′^ do not exhibit any specific behavior, we decided to disregard the increase in the cutoff radius. As shown in Figures 27, 28, 29 and 30:

### 3.3. Sensitivity Analysis of Models to Parameter *h*

To evaluate the responsiveness of electrophysiological models to variations in the Richards activation function, a sensitivity analysis was conducted for the parameter *h*. In this analysis, the optimal value of *h* was set to 0.7, and the resulting changes in key action potential metrics, including APA and APD, were examined.

For models simulating sodium channel dysfunction in BrS Type II, sensitivity was assessed based on changes in APA (measured in millivolts). Conversely, for models related to potassium channel dysfunction in BrS Type I, sensitivity was reported as changes in action potential duration (measured in milliseconds).

The results revealed that the PB and simplified models exhibited the greatest sensitivity to variations in parameter *h* in the potassium channel component, with APD changes of approximately 40.13 ms and 26 ms, respectively. On the other hand, the LR model showed the largest variation in V amplitude within the sodium channel, approximately 8.88 mV.

Furthermore, employing the Richards function with a tunable parameter, as opposed to classical functions such as the logistic function, provided greater flexibility in modeling the nonlinear behavior of ion channels. This feature is especially important in representing dysfunctions caused by genetic mutations.

The findings of this study align with recent advances in multiscale modeling and gene‐targeted therapies such as CRISPR [33–35].

Overall, the combined application of mathematical modeling, flexible nonlinear functions, and intelligent optimization algorithms provides a practical framework for developing personalized cardiac models for research and analysis, representing a significant step toward genetics‐based investigation of cardiac disorders.

In this study, the radius of Coulombic and van der Waals interactions were considered to be 1.2 nm. Additionally, to investigate sodium ion channel function under physiological conditions, an electrical potential difference of approximately 100 mV was applied across the channel.

## 4. Discussion

BrS is a complex cardiac channelopathy, most commonly associated with loss‐of‐function mutations in the SCN5A gene encoding the Nav1.5 sodium channel, and in some cases with dysfunction in potassium channels. At the cellular level, BrS phenotypes are generally linked to an imbalance between inward and outward ionic currents, particularly reduced INa and/or increased outward repolarizing currents, which can promote electrical instability and increase susceptibility to malignant ventricular arrhythmias. Therefore, investigating how sodium channel activation dynamics and current profiles influence AP characteristics provides a relevant computational framework for understanding BrS‐related electrophysiological behavior.

In this study, we developed an electrophysiological modeling approach based on replacing the classical activation function with a nonlinear Richards activation function and optimizing its shape parameter *h* using the PSO algorithm. The main motivation for using this formulation was to introduce a flexible mathematical structure capable of capturing abrupt or nonlinear changes in V‐dependent gating profiles, which is important when modeling pathological conditions such as BrS. The results demonstrated that tuning the parameter *h* improved key AP features, including APA, APD, and t_peak, thereby producing waveforms that are more consistent with physiological profiles under both normal and pathological conditions. The use of PSO for fine‐tuning the parameter *h* enhanced the model′s adaptability to physiological characteristics, and the overall findings are consistent with recent studies employing optimization algorithms and data‐driven approaches in cardiac modeling and personalized electrophysiology. [33, 36, 37]

A comparative analysis across different electrophysiological models further indicated that the LR and PB models exhibited higher sensitivity to changes in the parameter *h*, suggesting that these models may be better suited for detailed analysis of sodium and potassium channel‐related dysfunctions in BrS. In addition, the sensitivity results showed that in most models, variations in parameter h had a stronger impact on potassium channel‐related components, consistent with the critical role of repolarizing currents in shaping AP duration and morphology. This highlights the importance of considering both sodium‐ and potassium‐driven mechanisms when simulating BrS phenotypes.

In parallel with the cellular‐level simulations, MD simulations were performed to investigate sodium ion behavior within the channel selectivity filter under different electrolyte conditions. The MD results indicated that ionic strength (I = 0.14 M) contributes to structural stability, as supported by RMSD analysis, but also introduces localized electrostatic effects that can influence ion conduction behavior. Under physiological ionic strength, the presence of additional ions generated localized electric fields that initially weakened effective ion transport across the channel at an applied transmembrane potential of 100 mV, resulting in disrupted ion flux. Increasing the applied potential difference to 200 mV alone did not fully restore effective ion conduction, suggesting that ion transport in the selectivity filter is not solely determined by the applied field strength but is also affected by simulation and interaction parameters governing short‐ and long‐range forces.

To further examine these effects, the cutoff radius for Coulombic and van der Waals interactions was increased from 1.2 to 1.4 nm, which successfully reactivated sodium ion conduction through the channel. Under these conditions, ion transport was restored via the natural “knock‐on” mechanism, where the entry of a new ion into the filter promotes the exit of the preceding ion, enabling chain‐like transfer. Additional simulations across a wider range of transmembrane potentials (−100 to 200 mV) and cutoff radii (1.2–1.6 nm), with and without ionic strength, confirmed that the combination of 200 mV, 1.4 nm cutoff radius, and 0.14 M ionic strength provided the most consistent ion conduction behavior.

Importantly, these molecular‐scale observations provide mechanistic insight into how ionic environment and electrostatic interactions can modulate sodium ion transport, which is a key determinant of functional INa at the cellular level. Although the electrophysiological simulations and MD simulations operate at different scales, together they support a coherent interpretation in which alterations in sodium channel conduction and gating behavior, driven by genetic factors and biophysical conditions, can translate into measurable changes in AP morphology and indices relevant to BrS. This multiscale perspective strengthens the interpretation of the computational results and supports the proposed approach as a useful platform for studying sodium channel dysfunction and BrS‐related electrophysiological consequences.

Additionally, the use of the Richards function with an adjustable parameter, compared to classical functions such as the logistic function, provides greater flexibility in modeling the nonlinear behavior of ion channels. This feature is especially important for representing dysfunctions caused by genetic mutations. The current study’s results are consistent with recent advancements in multiscale modeling and gene‐based therapeutic perspectives, including CRISPR‐related approaches. [33–35]

Overall, the combined application of mathematical modeling, flexible nonlinear functions, and intelligent optimization algorithms provides a practical framework for developing personalized cardiac models for research and analysis, representing a significant step toward genetically informed investigation of cardiac disorders.

## 5. Limitations

Despite the promising results, this study has several limitations. First, the electrophysiological simulations were conducted using simplified cellular models, which, while computationally efficient and suitable for sensitivity analysis and parameter optimization, do not fully capture the complexity of detailed human ventricular cardiomyocyte electrophysiology. In particular, the models do not account for tissue‐level heterogeneity, RVOT‐specific effects, conduction velocity, or ECG correlates, which limits direct translational interpretation.

Second, the MD simulations focused on ion behavior within the selectivity filter under specific ionic strength and simulation settings, and did not explicitly model the full lipid membrane or long‐timescale conformational transitions associated with channel gating. The use of supraphysiological applied electric fields is another simplification that may affect quantitative extrapolation to physiological conditions. These assumptions should be considered when interpreting the MD results, which provide mechanistic insights at the molecular level rather than direct tissue‐level predictions.

In addition, this study does not include region‐specific or tissue‐level mechanisms that may contribute to BrS manifestations. Future studies may address these limitations by incorporating more detailed cardiomyocyte and tissue models, extending MD simulations to longer timescales and membrane‐embedded systems, and integrating additional experimental or patient‐specific data to further validate and personalize the proposed framework.

## 6. Conclusion

This study presented a computational framework combining electrophysiological modeling and MD simulations to investigate cardiac sodium channel behavior in the context of BrS. At the cellular level, replacing the classical activation function with a flexible Richards function and optimizing its shape parameter *h* using PSO improved key action potential features, including APA, APD, and t_peak, under both normal and pathological conditions. At the molecular level, MD simulations demonstrated that physiological ionic strength (I = 0.14 M) enhances structural stability but can initially disrupt sodium ion conduction through localized electrostatic effects; ion transport was restored by adjusting the applied transmembrane potential and the cutoff radius, reproducing the knock‐on mechanism. Overall, the proposed approach provides a practical and adaptable platform for simulating ion channel dysfunction and analyzing electrophysiological alterations relevant to BrS.

## Funding

No funding was received for this manuscript.

## Conflicts of Interest

The authors declare no conflicts of interest.

## Data Availability

The data which are used are referred to the corresponding references.
